# Development of Metal-Organic Framework-Based Dual Antibody Nanoparticles for the Highly Specific Capture and Gradual Release of Circulating Tumor Cells

**DOI:** 10.3389/fbioe.2022.806238

**Published:** 2022-02-07

**Authors:** Mingchao Hu, Cheng Li, Zhili Wang, Pi Ding, Renjun Pei, Qiang Wang, Hua Xu, Chungen Xing

**Affiliations:** ^1^ Department of Gastrointestinal Surgery, The Second Affiliated Hospital of Soochow University, Suzhou, China; ^2^ School of Nano-Tech and Nano-Bionics, University of Science and Technology of China, Hefei, China; ^3^ Department of General Surgery, The Affiliated Jiangsu Shengze Hospital of Nanjing Medical University, Suzhou, China

**Keywords:** circulating tumor cells, metal organic frameworks, isolation, cell release, layer-by-layer assembly method

## Abstract

Circulating tumor cells (CTCs) have been well-established as promising biomarkers that can be leveraged to gauge the prognosis of patients with cancers and to guide patient treatment efforts. Although the scarcity of CTCs within peripheral circulation and the associated phenotypic changes that they exhibit owing to the epithelial-mesenchymal transition (EMT) process make the reliable isolation of these cells very challenging. Recently, several studies have discussed platforms capable of mediating the efficient and sensitive isolation of CTCs, but these approaches are nonetheless subject to certain limitations that preclude their clinical application. For example, these platforms are poorly-suited to minimizing damage in the context of cellular capture and release or the *in vitro* culture of captured cells for subsequent molecular analyses, which would better enable clinicians to select appropriate precision treatments on an individualized basis. In this study, we report the layer-by-layer assembly approach to synthesize a novel composite nanomaterial consisting of modified zirconium-based metal-organic-frameworks (MOFs) on the surface of magnetic beads with dual antibody surface modifications capable of capturing CTCs without being hampered by the state of cellular EMT process. Our analyses indicated that these dual antibody-modified nanomaterials exhibited greater capture efficiency than that observed for single antibody. Importantly, captured cells can be gradually released following capture and undergo subsequent *in vitro* proliferation following water molecule-induced MOF structural collapse. This release mechanism, which does not require operator intervention, may be effective as a means of minimizing damage and preserving cellular viability such that cells can be more reliably utilized for downstream molecular analyses and associated treatment planning. To further confirm the potential clinical applicability of the developed nanomaterial, it was successfully utilized for capturing CTCs from peripheral blood samples collected from cases diagnosed with gastrointestinal tumors.

## Introduction

*Cancer* is one of the principal reasons for human morbidity and mortality globally. Despite notable advancements in the treatment and diagnosis of various cancers, metastatic disease progression remains a leading driver of patient death (Jolly et al., 2019). As tumors grow, malignant cells may travel through the leaky local vasculature and ultimately enter into systemic circulation, at which time they are referred to as circulating tumor cells (CTCs) (Joosse et al., 2015). Previous research indicates that the spreading and migration of these CTCs is an important contributor to metastatic tumor progression (Yap et al., 2014). These CTCs also offer value as biomarkers amenable to use in the early diagnosis of specific cancers (Baek et al., 2019; Tsai et al., 2019), the monitoring of patient treatment responses (Turano et al., 2019; Khattak et al., 2020; Rau et al., 2020; Reduzzi et al., 2020), and the gauging of patient prognosis (Yang et al., 2018; Trapp et al., 2019; Wang et al., 2019; Wang et al., 2020). These CTCs consist of cells taken from primary tumors or/and metastatic tissue sites that can be evaluated to gain comprehensive insights regarding the molecular status of the underlying disease, providing valuable feedback to inform clinical decision-making. However, these CTCs are scarce and heterogeneous regarding their molecular phenotypes, making their reliable isolation and identification challenging.

Several platforms have been advanced in recent decades that facilitate the isolation of CTCs based upon either the cellular physical characteristics or on specific cell surface antigens present on these CTCs (Nagrath et al., 2016). Physical characteristic-based cell capture methods include those based on cell size- (Vona et al., 2000; Oh et al., 2017), density- (Weitz et al., 1998; Rosenberg et al., 2002), inertia- (Warkiani et al., 2015), electrophoretic- (Gascoyne et al., 2009; Moon et al., 2011), and photoacoustic-based approaches (Galanzha and Zharov, 2013; Nedosekin et al., 2013). Immunoaffinity-based capture methods generally facilitate the isolation of CTCs through the binding of EpCAM, which is expressed at high levels on the surface of many such tumor cells. However, EpCAM is downregulated on CTCs undergoing the EMT process wherein epithelial cells acquire mesenchymal-like characteristics conducive to invasive, aggressive growth (Satelli et al., 2015; Wu et al., 2017; Liu et al., 2019b). Various recent investigations have shown that the EMT phenotype is closely tied to prognostic outcomes for a range of cancers (Satelli et al., 2015; Wu et al., 2017; Liu et al., 2019b; De et al., 2019; Nicolazzo et al., 2019; Yousefi et al., 2020). Developing a platform capable of simultaneously capturing CTCs exhibiting both mesenchymal and epithelial cell surface characteristics would offer an opportunity to better understand the mechanistic basis for tumor metastasis while offering new therapeutic targets and therapeutic options aimed at the effective treatment of cancers.

MOFs are crystalline porous hybrid nanomaterials consisted of metal clusters or ions joined by organic connectors (Ou et al., 2019). MOFs are widely employed for the catalytic purification of wastewater, gas adsorption, and separation applications (Furukawa et al., 2013; Zhang et al., 2018). As they are highly flexible and exhibit a very large surface area with extensive internal porosity, MOFs have also drawn the interest of researchers in the fields of biosensing (He et al., 2014a; Dong et al., 2018; Wang et al., 2018; Afreen et al., 2020), cancer treatment (He et al., 2014b; Chen et al., 2017), and drug delivery (Horcajada et al., 2010; Della Rocca et al., 2011). Several recent studies have sought to employ MOFs as tools to enhance the efficiency and purity of CTC capture. For example, in one recent report, researchers detected MCF-7 tumor cells using a MOFTA sensor constructed from PCN-224 and DNA tetrahedron coupled to dual aptamers (AS1411 & MUC1) (Ou et al., 2019). In a separate study, researchers proposed to design of a core-shell-based MOF nanoparticle that had been modified with an EpCAM-specific antibody to facilitate the capture and release of CTCs (Xie et al., 2019). However, most MOFs are limited by their poor stability in water, with their structures being susceptible to collapse as a consequence of the slow substitution of metal-ligand linkers by water molecules (Burtch et al., 2014; Yuan et al., 2018; Ding et al., 2019). Zr-based MOFs represent the most thermally and chemically stable MOFs produced to date (Cavka et al., 2008), and the Zr-based MOF UIO-67 has been repeatedly shown to exhibit good stability in aqueous solutions (Zhu et al., 2015; Øien-Ødegaard et al., 2016; Pankajakshan et al., 2018). In a previous report, we developed a dual antibody-based CTC capture platform with specificity for both N-cadherin and EpCAM, thereby facilitating the highly efficient isolation of CTCs with both epithelial and mesenchymal characteristics (Liu et al., 2019a). Here, we employed a layer-by-layer approach to generate UIO-67-modified Fe_3_O_4_ nanoparticles that underwent further dual antibody modification to facilitate the recognition of CTCs irrespective of EMT state. The generated composite nanomaterial was able to capture cells and then gradually release them for subsequent *in vitro* culture following the collapse of this structure upon water exposure.

## Experimental Section

### Materials and Reagents

Ferric chloride hexahydrate (FeCl_3_.6H_2_O), trisodium citrate (TSC), bovine serum albumin (BSA), polyvinyl pyrrolidone (PVP, Mw: 10,000), Zirconium(IV) chloride (ZrCl_4_), 1,1′-biphenyl-4,4′-dicarboxylic acid (BPDC), Streptavidin (SA), carbodiimide (EDC), N-hydroxysuccinimide (NHS) were provided from Sigma Aldrich (MO, United States). Polyethylene glycol (PEG, MW: 4000) was acquired from Aladdin Co., Ltd. (Shanghai, China), sodium acetate trihydrate (NaAc·3H_2_O), ethylene glycol (EG), N,N-Dimethylformamide (DMF) were provided from Sinopharm Chemical Reagent Co., Ltd. (Shanghai, China), Hoechst 33342, AF488-conjugated CD45 mouse mAb, AF555-conjugated Pan-Keratin mouse mAb, 1,1′-dioctadecyl-3,3,3′,3′-tetramethylindo-carbocyanine perchlorate (DiI), 3-3′-dioctadecyloxa-carbocyanine perchlorate (DiO) were procured from Cell Signaling Technology, Inc. (Danvers, MA). Biotinylated antibodies specific for human EpCAM and N-cadherin were obtained from Univ-bio Co. Ltd. (Shanghai, China).

### Fe_3_O_4_ Nanoparticle Synthesis

A solvothermal method was used to synthesize Fe_3_O_4_ nanoparticles as reported previously (Guo et al., 2016). Briefly, FeCl_3_.6H_2_O (0.945 g, 3.50mmool), PEG (0.50 g, 0.125 mmol), and TSC (0.70 g, 2.38 mmol) were combined with gentle mixing in 70 ml of EG solution until the solvent was fully dissolved to yield a transparent solution, after which NaAc·3H_2_O (6.96 g, 51.15 mmol) was added and the mixture was stirred vigorously at ambient temperature for 1 h. The mixture was then poured into a reactor and heated to 200°C for 10 h, following which the obtained product was washed thrice in anhydrous ethanol, dissolved in anhydrous ethanol, and stored at 4°C.

### Dual-Antibody-Fe_3_O_4_@UIO-67 Synthesis

A layer-by-layer method was used to synthesize core-shell structure nanoparticles. Briefly, nanoparticles were prepared by dispersing 50 mg of Fe_3_O_4_ in 50 ml of 10 mg/ml PVP aqueous solution and stirring the resultant mixture for 24 h at ambient temperature. The resultant product was collected, rinsed thrice with anhydrous ethanol, added to 20 ml of DMF along with 0.2 mmol of ZrCl_4,_ and stirred at ambient temperature for 30 min. The product was then collected *via* magnetic separation, rinsed using DMF, and nanoparticles were then dispersant in 20 ml of 10 mmol/L BPDC DMF solution and heated for 40 min at 70°C followed by an additional wash with DMF. This procedure was repeated five times to achieve layer-by-layer assembly, yielding UIO67-coated Fe_3_O_4_ particles hereafter referred to as Fe_3_O_4_@UIO-67. Reaction product concentrations were measured, dissolved with anhydrous ethanol, and stored at 4 °C for subsequent utilization.

Next, antibody surface modification was achieved by washing 200 μg of Fe_3_O_4_@UIO-67 with PBS three times and transferring them into 0.1 M MES buffer containing 0.025 M NHS and 0.1 M EDC with shaking at 37°C for 30 min. The resultant products were then magnetically separated, rinsed thrice with PBS, and then suspended in 900 uL of PBS and 100 ul of SA solution (20 μg/ml in PBS) with gentle shaking at ambient temperature for 10 h. Samples were again rinsed thrice with PBS, yielding SA-modified particles that were subsequently incubated at room temperature with appropriate biotinylated antibodies (2 μg/ml anti-EpCAM or anti-N-cadherin in PBS) for 2 h with constant shaking. The resultant particles were again washed and then suspended in 2% (w/v) BSA for 2 h with constant shaking to prevent non-specific binding to the prepared composite nanomaterial.

### Dual Antibody-Modified Fe3O4@UIO-67 Characterization

Fe_3_O_4_@UIO-67 morphological characteristics were identified *via* transmission electron microscopy (TEM) employing a Hitachi-HT7700 instrument (accelerating voltage: 120 kV). Fe_3_O_4_@UIO-67 particle size was assessed by implementing a Dynamic Light Scattering (DLS) instrument (Zetasizer Nano ZS ZEN3600, Malvern Instruments Ltd. United Kingdom). Following layer-by-layer assembly, composite nanomaterial surface morphology was additionally assessed *via* scanning electron microscopy (SEM, 20.0 kV, FEI Quanta 400F).

### Cell Culture

The human MCF-7 breast cancer (EpCAM+, N-cadherin-), HeLa cervical cancer (EpCAM-, N-cadherin+), and CCRF-CEM acute lymphoblastic leukemia T lymphocyte (EpCAM-, N-cadherin-) cell lines were used as models in the present study. HeLa and MCF-7 cells were cultivated in DMEM comprising 1% penicillin/streptomycin and 10% fetal bovine serum (FBS), while CCRF-CEM cells were cultured in RPMI-1640 containing 1% penicillin/streptomycin and 10% FBS. All cells were cultivated in a 5% CO_2_ 37°C incubator, with media being replaced every other day. Prior to cell capture assays, cells were harvested employing 0.05% trypsin.

### Antibody-Functionalized Fe_3_O_4_@UIO-67-Mediated Cell Capture Studies

A hemocytometer was used to count model cells, which were then combined with antibody-modified composite nanomaterials at experimentally appropriate concentrations for 30 min at ambient temperature to assess cell capture dynamics. Capture efficiency was determined as the ratio of the total number of cells captured by the prepared nanomaterial to the starting cell number. Analyses were repeated in triplicate, with results being reported as the mean ± standard deviation. Associations between co-incubation time and cell capture efficiency for prepared composite nanomaterials, 100,000 MCF-7 cells were incubated with anti-EpCAM-modified magnetic beads for 10, 15, 20, or 25 min, after which samples were magnetically separated, washed thrice with PBS, and cell capture efficiency was calculated. The sensitivity of dual antibody-modified Fe_3_O_4_@UIO-67 particles as a tool for capturing low numbers of CTCs, 20, 50, 100, or 200 HeLa or MCF-7 cells that had been pre-stained by utilizing Dio dye were suspended in 1 ml of PBS or healthy human donor blood to simulate peripheral blood samples from cancer patients. Following a 30 min co-incubation at 37°C, samples were magnetically separated, rinsed with PBS, and capture efficiency was computed.

### Culture and Proliferation Analyses of Captured CTCs

After cells had been captured using modified nanoparticle preparations, they were rinsed thrice with PBS and added to DMEM for subsequent culture under standard conditions as described above. Cellular fluorescence was assessed by staining cells with the Dio dye after 24, 48, 72, or 96 h. In addition, a CCK-8 assay was used based on provided protocols to assess cellular proliferation, while cellular viability was quantified with a Calcein-AM/PI Double Stain Kit. Briefly, 50,000 HeLa cells were incubated for 30 min with dual antibody-modified Fe_3_O_4_@UIO-67 at 37 °C, after which samples were rinsed with PBS and captured cells were stained with 2 μM calcein-AM (green) and 4.5 μM PI (red) for 20 min at 37°C. After a subsequent rinse with PBS, cells were imaged *via* fluorescence microscopy, and the ImageJ application was employed for counting dead (red) and live (green) cells. All analyses were repeated in triplicate.

### CTC Capture From Cancer Patient Peripheral Blood Samples

Samples of whole peripheral blood of cancer patients were accumulated from the Second Affiliated Hospital of Soochow University and The Affiliated Jiangsu Shengze Hospital of Nanjing Medical University in tubes comprising Ethylene Diamine Tetraacetic Acid (EDTA) as an anticoagulant. The ethics committees of the participating hospitals approved all aspects of the present study. All samples were processed within 48 h following collection. In total, 5 ml of peripheral blood was collected from each of 10 cancer patients. Each sample was then diluted to a 10 ml total volume using HBSS (Hank’s balanced salt solution) and layered atoll 5 ml of Ficoll-Paque (Sigma-Aldrich), after which peripheral blood mononuclear cells (PBMCs) were isolated through centrifuging samples at ambient temperature for 30 min at 2,000 rpm, rinsing the interface later with HBSS three times, and then incubating samples for 30 min with dual antibody-functionalized magnetic beads at 37°C. Captured cells were then washed with PBS, fixed with 4% paraformaldehyde for 20 min, and suspended for 1 h in blocking buffer (1× PBS with 5% FBS and 0.3% Triton X-100). CTCs were identified with Alexa Fluor 555-conjugated anti-PanCK, while leukocytes were identified using Alexa Fluor 488-conjugated anti-CD45, with Hoechst 33342 being used for nuclear counterstaining. Cells were then imaged *via* confocal microscopy, enabling the differentiation between CTCs (CK+/CD45-/Hoechest+) and leukocytes (CK-/CD45+/Hoechest+).

## Results

### Fe_3_O_4_@UIO-67 Preparation and Characterization

**FIGURE 1:**
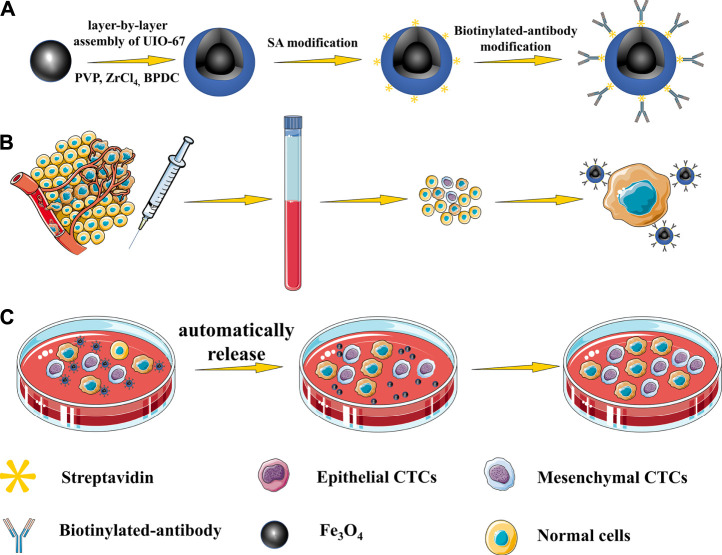
**(A)** Nanoparticles with a core-shell structure were prepared via a layer-by-layer method. **(B)** Highly specific CTC capture approach using dual antibody-modified nanoparticles. **(C)** UIO-67 gradually collapses in aqueous solutions, thereby facilitating the gradual automated release of captured cells.

**FIGURE 2:**
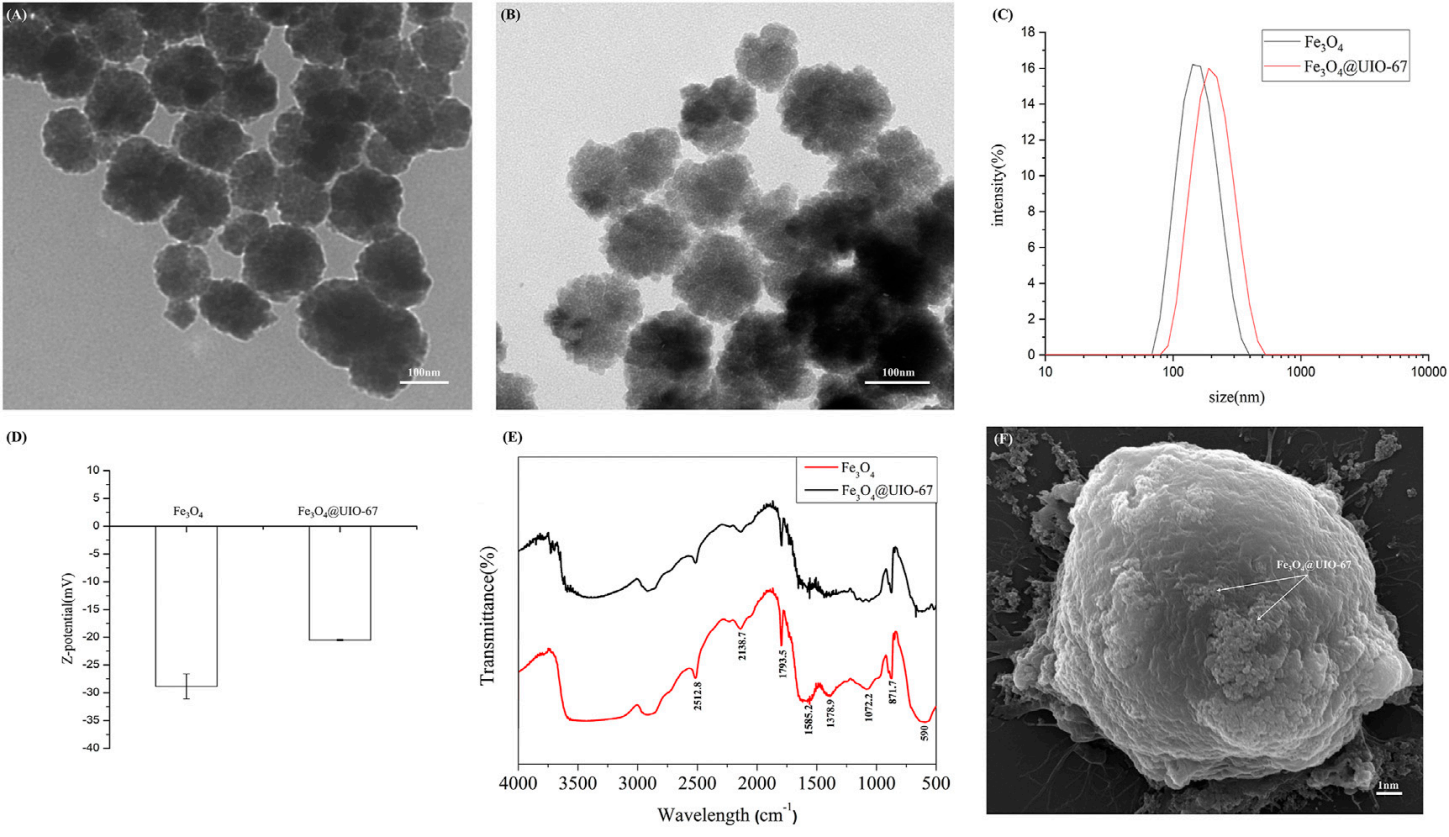
**(A)** Images of TEM for pure Fe_3_O_4_. **(B)** Images of TEM for Fe_3_O_4_@UIO-67. **(C)** Hydrodynamic diameter values for Fe_3_O_4_ and Fe_3_O_4_@UIO-67 preparations measured *via* DLS. **(D)** Zeta potential values for Fe_3_O_4_ and Fe_3_O_4_@UIO-67 preparations. **(E)** Fe_3_O_4_ and Fe_3_O_4_@UIO-67 FTIR spectra. **(F)** Representative MCF-7 cell images following capture using dual antibody-modified Fe_3_O_4_@UIO-67.

We began by synthesizing Fe_3_O_4_ *via* a hydrothermal method, after which UIO-67 was used to modify the surface of these particles *via* a layer-by-layer assembly approach to yield a composite nanomaterial with a stable core-shell structure as shown in Figure 1. The surface morphology of these prepared Fe_3_O_4_ and Fe_3_O_4_@UIO-67 particles was assessed *via* TEM (Figure 2A,B), while DLS was employed for confirming the size and Zeta potential of these particles following UIO-67 modification. The diameters of unmodified Fe_3_O_4_ and UIO-67-modified Fe_3_O_4_ in this analysis were 169.90 ± 9.37 nm and 231.91 ± 16.22 nm, respectively, which indicated that UIO-67 was successfully modified on the surface of the Fe_3_O_4_ using the layer-by-layer assembly method (Figure 2C). Zeta potential values indicated that while Fe_3_O_4_@UIO-67 is relatively stable in water, it is more labile than Fe_3_O_4_ (Figure 2D). The gradual collapse of the prepared MOF structure in aqueous solutions would thus have the capability to facilitate the release of captured CTCs for subsequent culture and analysis. In Fourier-transform infrared (FTIR) spectra analyses of Fe_3_O_4_, the absorption peak at 590 cm^−1^ was attributed to the stretching vibration of the Fe-O functional group, while the peak at 1,585.2 cm^−1^ corresponded to carboxyl group vibrations as a consequence of the red-shift of the carboxyl peak when the carboxylic acid ligand interacts with the metal ion in the context of coordination, thus reducing its energy and increasing associated stability (Figure 2E). The peak at 871.7 cm^−1^ was further assigned to the flexural vibration of C-H within the benzene ring. Comparisons of the FTIR spectra for pure Fe_3_O_4_ and the prepared composite nanoparticles confirmed that UIO-67 had successfully been used to modify the surfaces of these Fe_3_O_4_ nanoparticles. Representative images of SEM for MCF-7 cells captured using dual antibody-modified versions of these composite nanoparticles are shown in Figure 2F, demonstrating that many nanoparticles were visible on captured cell surfaces.

### Cell Capture Condition Optimization

**FIGURE 3:**
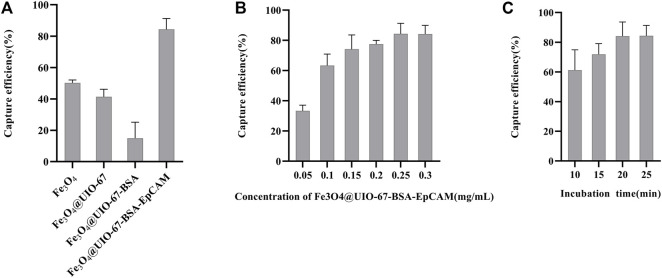
**(A)** Analysis of the nanoparticle modification status on MCF-7 cell capture efficiency. **(B)** Analysis of the effects of nanoparticle concentration on MCF-7 cell capture efficiency. **(C)** Analysis of the effects of time on MCF-7 cell capture efficiency.

**FIGURE 4:**
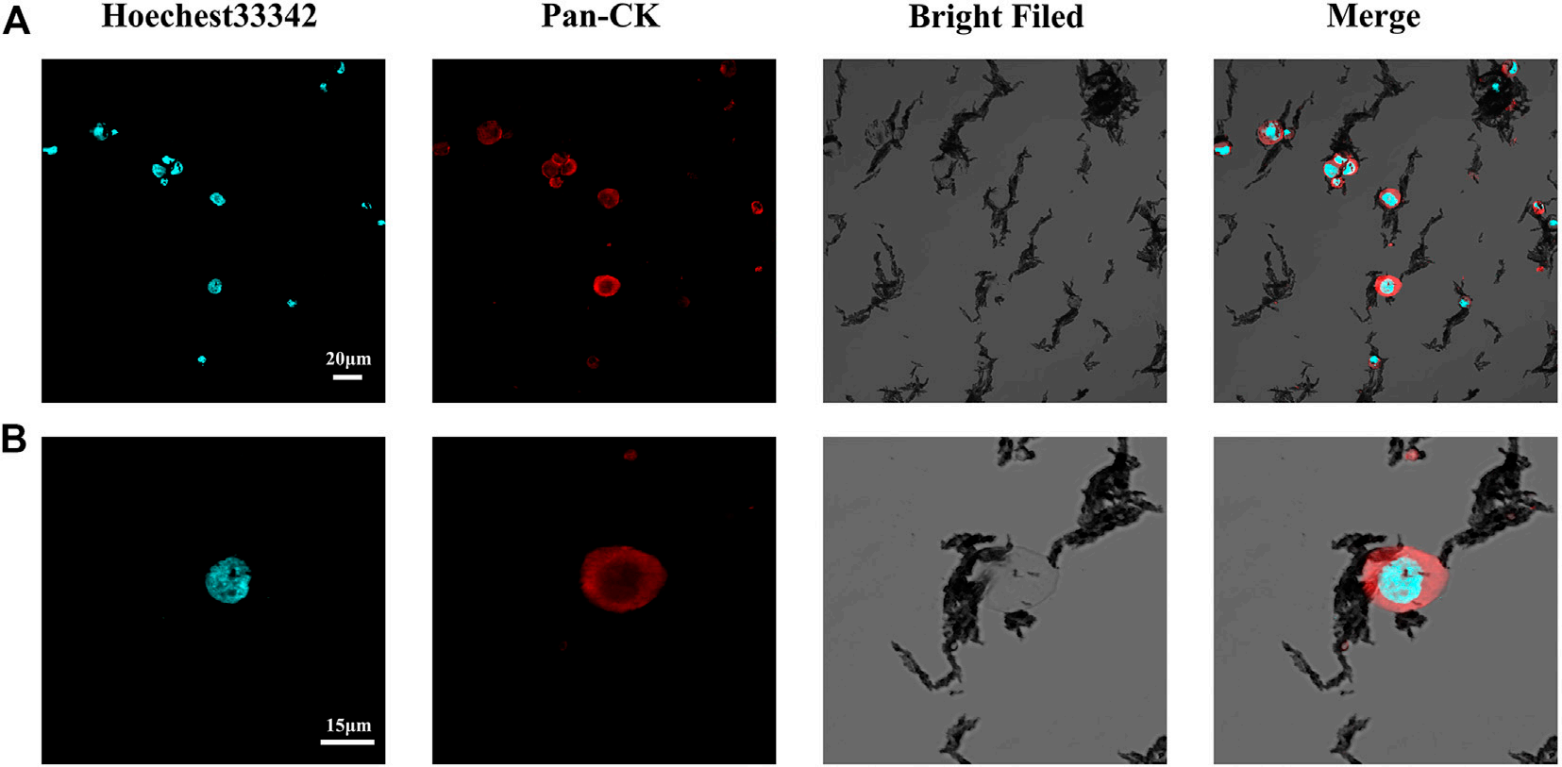
**(A)** Representative confocal microscopy images of captured MCF-7 cells. **(B)** Representative confocal microscopy images of single captured MCF-7 cells.

To verify the effective capture of target tumor cells employing our dual antibody-modified Fe_3_O_4_@UIO-67 platform, we next sought to optimize cell capture conditions. To that end, we utilized MCF-7 tumor cells expressing high levels of EpCAM to mimic tumor cells with an epithelial-like phenotype, incubating 10^5^ MCF-7 cells with different nanoparticle preparations for 40 min. Samples were then magnetically separated and washed three times, after which capture efficiency was calculated. Modification of UIO-67 using layer-by-layer assembly on these particles did not effectively eliminate non-specific binding in this assay context (Figure 3A), leading us to next block the surface of the prepared nanoparticles with 2% (w/v) BSA for 2 h following dual antibody modification, thereby significantly reducing nonspecific cellular adhesion. We next explored the impact of nanoparticle concentrations and incubation duration on the efficiency of MCF-7 capture using our anti-EpCAM-modified nanoparticles, revealing that capture efficiency rose with nanoparticle concentration to a maximum of 84.375 ± 6.884% at a nanoparticle concentration of 0.25 mg/ml (Figure 3B). Capture efficiency was also influenced by incubation time, with maximal efficiency being achieved following a 20 min incubation (Figure 3C). Representative fluorescent images of MCF-7 cells following dual antibody-modified nanoparticle-mediated capture are shown in Figure 4A,B, with isolated MCF-7 cells exhibiting red fluorescence attributable to anti-Pan-CK staining.

### Evaluation of the Impact of Modification Status on the UIO-67-Coated Magnetic Nanoparticle Cell Capture Efficiency

**FIGURE 5:**
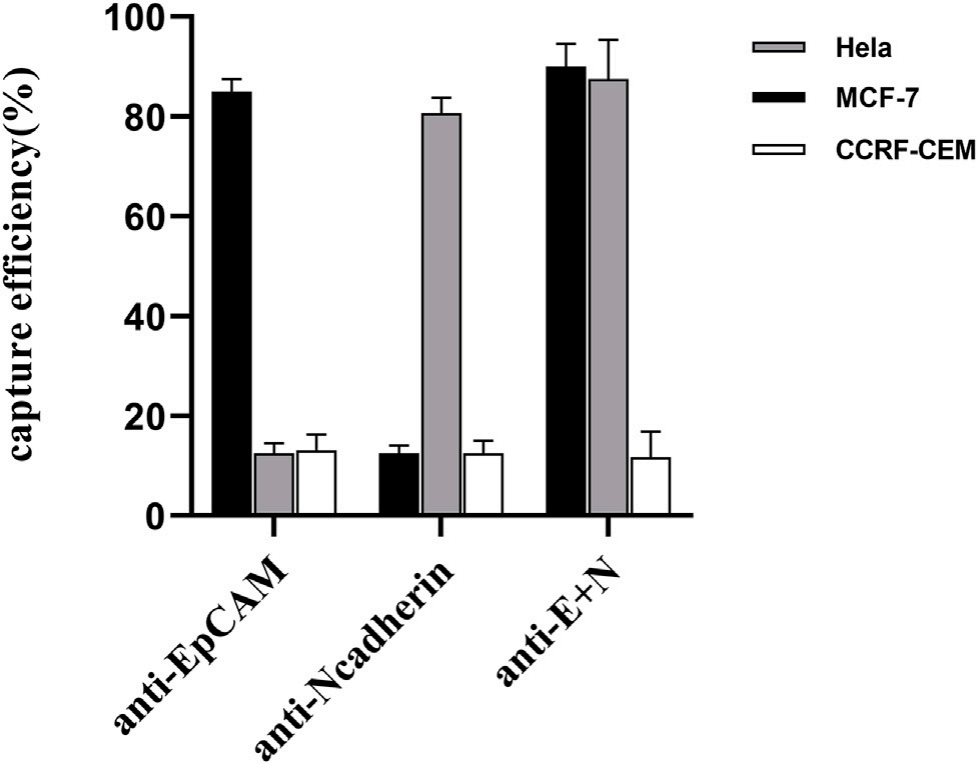
Comparing the rates of capture efficiency when using anti-EpCAM-modified, anti-N-cadherin-modified, and dual antibody-modified nanoparticles to capture model target cell lines.

We additionally explored the ability of nanoparticles modified with different antibodies to capture simulated CTCs exhibiting different surface phenotypes, with MCF-7 (EpCAM+, N-cadherin-) and HeLa (EpCAM-, N-cadherin+) cells being used to simulate CTCs with epithelial and mesenchymal characteristics, respectively. In addition, CCRF-CEM cells were used to evaluate non-specific nanoparticle cell binding as these cells do not express EpCAM or N-cadherin. Prepared anti-EpCAM-modified nanoparticles exhibited high capture efficiency values for MCF-7 cells (83.75 ± 1.25%) but not for HeLa (12.75 ± 1.79%) or CCRF-CEM (13.125 ± 2.724%) cells, consistent with the feasibility of this specific CTC capture approach (Figure 5). Similarly, anti-N-cadherin-modified nanoparticles exhibited high capture efficiency for HeLa cells (80.625 ± 2.724%) but not for MCF-7 or CCRF-CEM cells (2.5 ± 1.44% and 12.5 ± 2.04%, respectively). Dual antibody-modified nanoparticles were also able to more efficiently capture both MCF-7 (90 ± 3.95%) and HeLa (87.5 ± 6.85%) cells as compared to single antibody-modified nanoparticles, consistent with the ability of these nanoparticles to capture CTCs irrespective of EMT progression status.

### Analysis of Rare Cell Capture Efficiency

**FIGURE 6:**
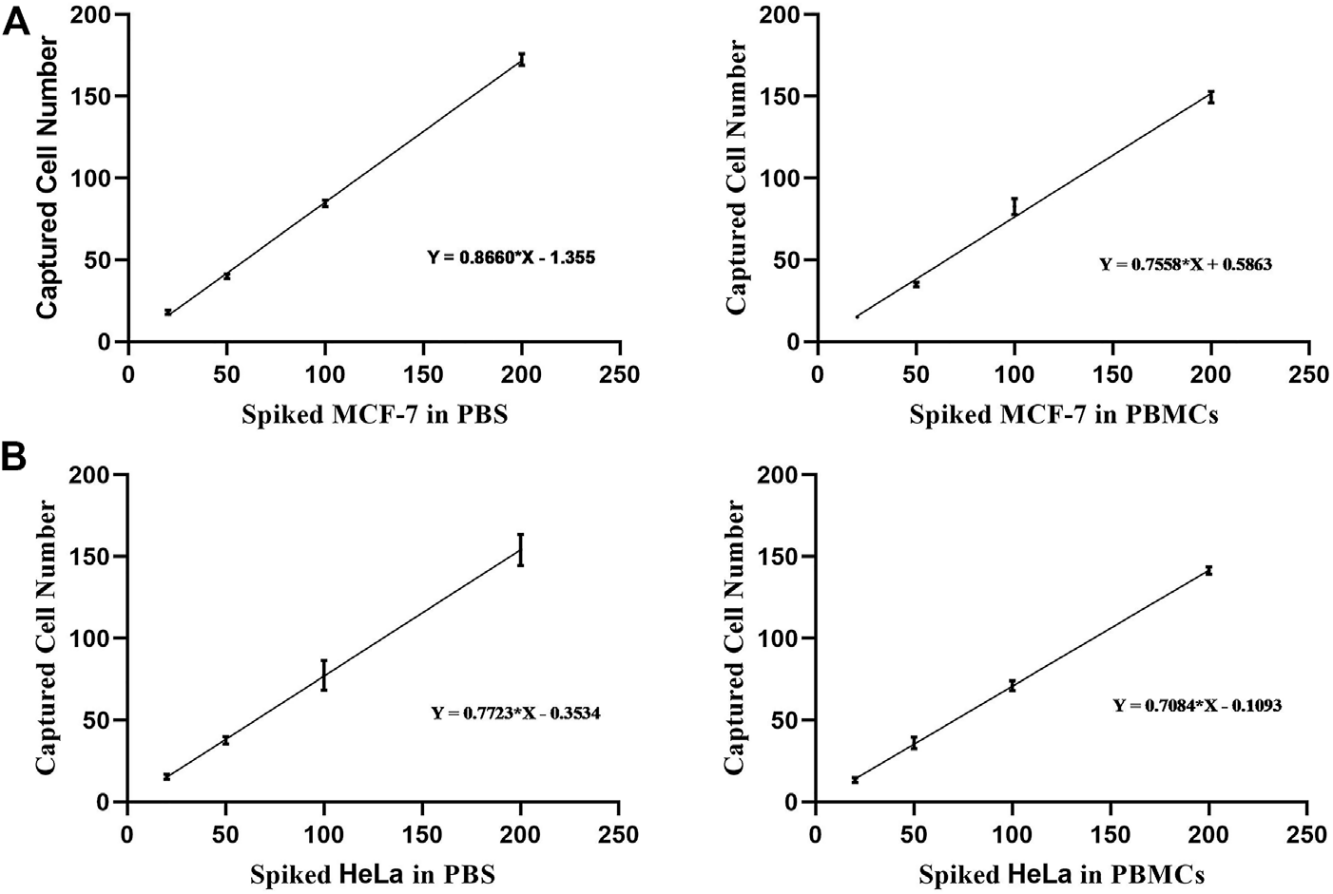
**(A)** Analysis of the capture efficiency for low numbers of MCF-7 cells in PBS or PBMC-containing solutions. **(B)** Analysis of the capture efficiency for low numbers of HeLa cells in PBS or PBMC-containing solutions.

Prior experimental studies suggest that dual antibody-modified nanoparticles that had been blocked with BSA (2%, w/v) were well-suited to the efficient capture of target cells of interest with minimal non-specific adsorption. To further confirm the specificity of the developed call capture platform, we sought to assess the ability of our modified composite nanomaterial to detect rare simulated CTCs by adding low numbers of Dio-labeled MCF-7 or HeLa cells (20, 50, 100, or 200 cells) in 1 ml of PBS. Subsequent analyses indicated that over 86% of MCF-7 cells and 77% of HeLa cells were captured through prepared dual antibody-modified nanoparticles under these conditions (Figure 6A). We then repeated these experiments following the addition of Dio-stained tumor cells to healthy donor PBMCs to better mimic the complex milieu present within peripheral blood-derived samples gathered from cancer patients. Under these conditions, the capture efficiency rates for MCF-7 and HeLa cells declined slightly to 75 and 70%, respectively, likely owing to the interference stemming from the large numbers of background cells present within these whole blood-derived PBMC samples.

### Analysis of Captured Cell Release and Viability

**FIGURE 7:**
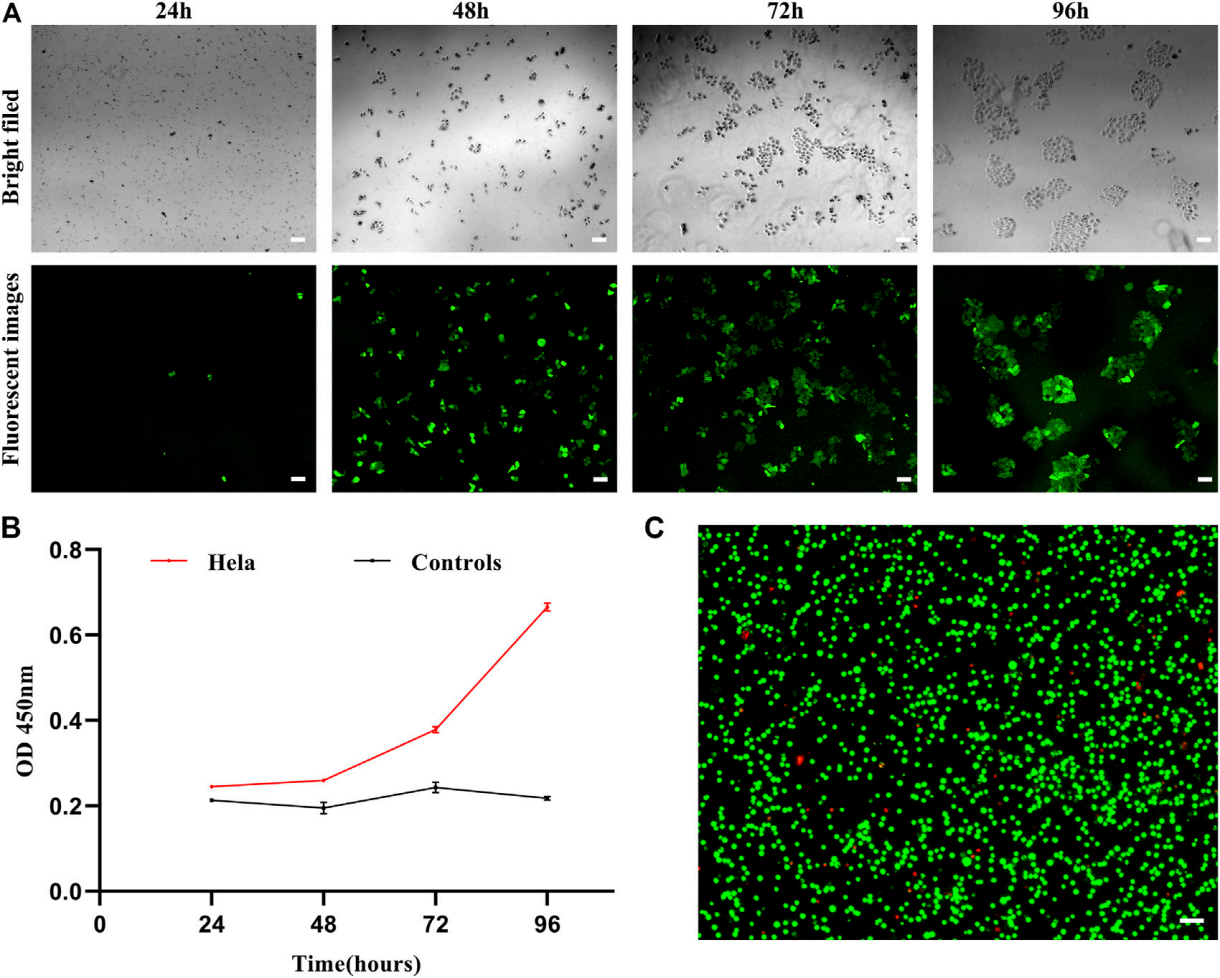
**(A)** Brightfield and fluorescent images of HeLa cells following dual antibody-modified nanoparticle-mediated capture and subsequent culture for 24, 48, 72, or 96 h. **(B)** Absorbance (OD) at 450 nm for HeLa cell cultures following dual antibody-modified nanoparticle-mediated capture. **(C)** Fluorescent imaging of HeLa cell viability following nanoparticle-mediated capture. Scale bar: 100 μm.

Given their rarity, the ability to transiently capture CTCs *in vitro* and to subsequently culture them for use in subsequent mutational or gene expression analyses would be invaluable as a means of better guiding patient care and more fully understanding the mechanisms shaping tumor pathogenesis. As such, we next co-cultured captured cells and nanoparticles *in vitro* and then evaluated the viability and proliferation of cells over time. This analysis revealed that after an initial quiescent period, these captured cells were able to proliferate in culture (Figure 7A,B), consistent with their gradual release from the MOF structure as it collapses in water. This progressive dissolution of the MOF structure following interaction with water can thus facilitate the automated release of captured cells in a non-damaging manner, thus enabling their subsequent proliferation and *in vitro* culture for downstream molecular analyses while maintaining high levels of viability (Figure 7C).

### Dual Antibody-Modified Fe_3_O_4_@UIO-67-Mediated Capture of Peripheral Blood CTCs From Gastrointestinal Cancer Patients

**FIGURE 8:**
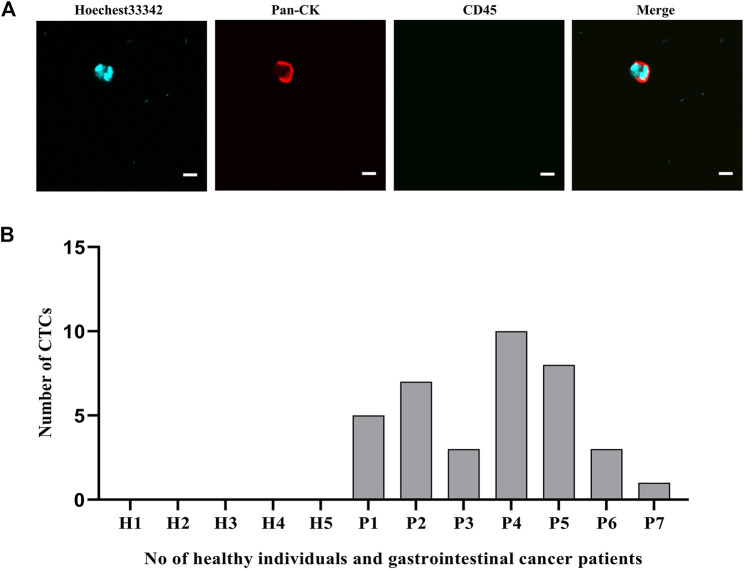
**(A)** Representative fluorescent images of captured CTCs. **(B)** Quantification of the CTCs captured from the peripheral blood (5 ml) of patients with gastrointestinal tumors. Scale bar: 20 μm.

To confirm the potential clinical viability of these dual antibody-modified nanoparticles, we next utilized them in an effort for capturing CTCs from the peripheral blood of gastrointestinal cancer cases (n = 7) and healthy controls (n = 5) using blood samples collected from the Second Affiliated Hospital of Soochow University and The Affiliated Jiangsu Shengze Hospital of Nanjing Medical University. Following nanoparticle-mediated CTC capture, immunofluorescent staining was conducted to differentiate between leukocytes (CK-/CD45+/Hoechest+) and CTCs (CK+/CD45-/Hoechest33342+). Representative images of captured CRCs are shown in Figure 8A, with the quantification of the numbers of peripheral blood CTCs captured from each gastrointestinal cancer patient being shown in Figure 8B. On average, 1-10 CTCs were collected in the peripheral blood samples from these cancer cases, whereas 0 CTCs were evident in any of the healthy control blood samples. Together, these data thus demonstrate that these dual antibody-modified nanoparticles can reliably capture CTCs within the peripheral blood of cases harboring gastrointestinal tumors.

## Conclusion

In conclusion, we herein employed a layer-by-layer assembly approach to develop a novel composite nanomaterial *via* the modification of MOF structures on the surfaces of magnetic beads with dual-antibodies targeting epithelial and mesenchymal antigens expressed on the surfaces of CTCs. The constructed MOF structure underwent gradual collapse in water, thereby facilitating the gradual release of captured cells which can then undergo subsequent proliferation in culture. This composite nanomaterial offers many advantages include ease-of-synthesis, a low cost, and excellent biocompatibility, and its ability to capture viable CTCs for subsequent culture makes it amenable to use in the context of a variety of downstream molecular analyses.

## Data Availability

The raw data supporting the conclusions of this article will be made available by the authors, without undue reservation.
